# Higher interferon score and normal complement levels may identify a distinct clinical subset in children with systemic lupus erythematosus

**DOI:** 10.1186/s13075-020-02161-8

**Published:** 2020-04-25

**Authors:** Alessandra Tesser, Luciana Martins de Carvalho, Paula Sandrin-Garcia, Alessia Pin, Serena Pastore, Andrea Taddio, Luciana Rodrigues Roberti, Rosane Gomes de Paula Queiroz, Virginia Paes Leme Ferriani, Sergio Crovella, Alberto Tommasini

**Affiliations:** 1 Department of Pediatrics, Institute for Maternal and Child Health - IRCCS “Burlo Garofolo”, Trieste, Italy; 2 Ribeirão Preto Medical School, University of São Paulo, Ribeirão Preto, Brazil; 3 Federal University of Pernambuco, Recife, Brazil; 4 Department of Medicine, Surgery and Health Sciences, University of Trieste, Trieste, Italy

**Keywords:** Systemic lupus erythematosus, Interferon score, Complement, Autoinflammation, Autoimmunity, Patients’ stratification

## Abstract

**Background:**

Systemic lupus erythematosus (SLE) is a complex multi-system disease, characterized by both autoimmune and autoinflammatory clinical and laboratory features. The role of type I interferon (IFN) in SLE has been demonstrated from the 2000s, by gene expression analyses showing significant over-expression of genes related to type I IFN signalling pathway (IFN signature). However, several studies questioned the role of measuring the intensity of IFN signature (IFN score) to chase SLE activity.

We would assess if the IFN signature can help the clinical and therapeutic stratification of patients with pediatric SLE.

**Methods:**

We measured the IFN score in peripheral whole blood from a series of subjects with childhood-onset SLE and correlated the results with clinical and laboratory parameters.

**Results:**

Thirty-one subjects were included in the study, among which the 87% displayed a positive IFN score. The only significant relation was found for high IFN score in subjects with normocomplementemia. No correlation was observed between IFN score and SLEDAI-2K, BILAG-2004 and SLICC. Patients with high IFN score and normal complement levels also presented lower anti-dsDNA antibodies.

**Conclusions:**

The integration between IFN signature analysis and complement levels may easily distinguish two groups of subjects, in which the autoimmune or autoinflammatory component of the disease seems to be prevalent.

## Introduction

### Systemic lupus erythematosus

Systemic lupus erythematosus (SLE) is a multisystem inflammatory and autoimmune disorder and it is characterized by a wide spectrum of clinical manifestations. Clinical involvement may greatly differ among individuals. Although all organs and tissues may be involved, skin rash, arthritis and renal and haematological impairment are the more typical clinical signs [1, 2].

Childhood-onset systemic lupus erythematosus (cSLE) [3] occurs before 18 years of age and presents a more severe clinical course than SLE developed in adult age [4, 5].

In recent years, significant progress has been achieved in the understanding of SLE, thanks to the identification of novel genetic variants associated with the disease, studies on murine models or findings involving gene expression and epigenetics. Genetic alterations leading to impaired function of the machinery devoted to clear apoptotic cells, waste nucleic acids and immune complexes play a significant role in both Mendelian and sporadic forms of SLE. The imbalance of these mechanisms results in the production of autoantibodies and in a dysregulated activation of inflammatory phenomena dominated by the secretion of type I interferons. Loss of lymphocyte tolerance may contribute to an amplification of autoimmune and inflammatory features, responsible for tissue damage [6–10].

In 2006, McGonagle and McDermot [11] suggested that in a pathogenic continuum between autoimmunity and autoinflammation, SLE may be set closer to the former mechanism, being marked prevalently by autoimmune nature. However, the subsequent identification of cases of familial SLE with prevalent autoinflammatory pathogenesis drove the attention on the latter mechanism as well [12]. Unravelling the different contribution of autoimmunity and autoinflammation in individuals with SLE may provide useful clues for therapeutic stratification with treatments targeted more to lymphocytes or interferons.

### SLE and type I interferons

The connection between lupus and interferon (IFN) dates to 1979, when high levels of IFN have been recovered in sera from patients with SLE [13]. This study paved the way to other researches highlighting the crucial role of type I IFN in the pathogenesis of the disease: the IFN abundance was consistently confirmed in both sera [14, 15] and tissues specimens from subjects with SLE, such as skin and synovial biopsies [16, 17]. Overall, these evidences allowed to consider SLE as the first non-infectious disease associated with an increased type I IFN production.

### SLE and type I interferon signature

Since the early 2000s, SLE knowledge has increased thanks to the improvement of gene expression/sequencing technologies. The first studies conducted on peripheral blood cells of patients with SLE have shown substantial differences between gene expression profiles of affected and healthy subjects, underling that the differentially expressed genes were related to type I IFN signalling pathway, the so-called interferon-stimulated genes (ISGs). The over-expression of ISGs was ever more considered as a shared feature of SLE and lupus-like disorders, leading to the introduction of the “IFN gene expression signature” concept [18–20].

The application of IFN signature analysis in the clinical practice helped to describe in 2011 a novel group of Mendelian disorders, called type I interferonopathies, which are due to impaired nucleic acid sensing and/or metabolism, leading to an over-production of IFN. This new classification was formulated by Yanick Crow, one of the leading experts in the study of Aicardi-Goutières syndrome (AGS), considering the clinical and laboratory overlaps with SLE, AGS and some congenital viral infections of the TORCH (toxoplasmosis, rubella, cytomegalovirus, herpes simplex) and HIV (human immunodeficiency virus) group [21, 22]. Even though the first “IFN signature” was defined in subjects with SLE, different sets of ISGs are currently taken into account to classify pathological conditions characterized by a type I IFN dysregulation (i.e. monogenic interferonopathies, dermatomyositis, SLE), to guide molecular diagnostics and to formulate targeted therapy approaches. For example, a set of six ISGs has been proposed by Crow and validated on IFN-driven monogenic inflammatory diseases as a golden standard of positivity [23–25].

Considering these evidences, the IFN signature was proposed as a useful biomarker to chase SLE activity, even if the correlation studies between ISGs expression and changes in disease activity have led to controversial results [26–28].

Given that a greater genetic contribution for rheumatologic disorders can be expected in pediatrics and monogenic interferonopathies can display a wide clinical spectrum, it is likely to assume that autoinflammatory mechanisms may be represented in pediatric SLE more than in adults. Thus, even if the assessment of IFN signature in adults with SLE failed to give clear clues to classify patients or to assess disease activity, we hypothesize that children with SLE may represent a more suitable population to study the potential of this assay. This study aims at assessing if the IFN signature analysis can contribute to identify subgroups of patients with paediatric SLE who may benefit from distinct treatments, based on the prevalent mechanisms involved in the disease pathogenesis.

## Methods

### Study design and subjects

We performed a cross-sectional study in cSLE attending Pediatric Rheumatology Clinic in the Clinical Hospital of Ribeirão Preto Medical School (Brazil). All patients fulfilled both American College Rheumatology (ACR) lupus criteria [29, 30] and Systemic Lupus International Collaborating Clinics Classification Criteria for Systemic Lupus Erythematosus (SLICC) [31] and with the disease onset before 18 years.

The clinical data were obtained from the patients’ medical records according to protocol for demographic, lupus-related clinical, laboratory and therapeutic data. The SLE Disease Activity Index 2000 (SLEDAI-2K) [32], British Isles Lupus Assessment Group Index 2004 (BILAG-2004) [33] and Systemic Lupus International Collaborating Clinics/American College of Rheumatology Damage Index (SLICC/ACR DI) [34] were used to evaluate the disease activity and cumulative damage, respectively.

The SLEDAI-2K index scores (range from 0 to 105) defined the activity categories: mild activity (SLEDAI < 6), moderate activity (SLEDAI 6–10) and high activity (SLEDAI ≥ 11) [35]. The BILAG-2004 index categorized the activity disease into five different levels from grades A to E. Grade A represented very active disease requiring immunosuppressive drugs and/or more than 20 mg of prednisolone or equivalent daily; grade B represented moderately active disease requiring lower doses of glucocorticoids, antimalarials or nonsteroidal anti-inflammatory drugs (NSAID); and grade C indicated mild stable disease, whilst grade D indicated that there is no current disease activity but that the system had previously been affected and grade E indicated no current or previous disease activity. A numerical scoring for the BILAG-2004 index was calculated (A = 12, B = 8, C = 1 and D/E = 0). The damage index used SLICC/ACR DI ranging from 0 to 47.

Laboratory assessment included complete blood cell count, urine analysis, urine protein creatinine ratio, complement levels (C3 and C4), anti-dsDNA and antiphospholipid (aPL) autoantibodies (lupus anticoagulant, anticardiolipin IgM and IgG and anti-β2 glycoprotein). Cut-off values were considered abnormal according to the assay manufacturer.

Current therapy (prednisone, intravenous methylprednisolone pulse, hydroxychloroquine sulphate, methotrexate, azathioprine, cyclosporine, mycophenolate, intravenous cyclophosphamide) was also recorded.

Ten young-aged healthy subjects were selected as representative of healthy populations (five males and five females) to perform the gene-expression analysis.

### RNA isolation and cDNA preparation

Peripheral blood was collected in EDTA. Total RNA was extracted with TRIzol® reagent (Thermo Fisher Scientific, USA) following the manufacturer’s instructions and quantified with NanoDrop 2000 Spectrophotometer (Thermo Fisher Scientific, USA).

Up to 500 ng of total RNA was retro-transcribed using MultiScribe™ Reverse Transcriptase (Thermo Fisher, USA), and cDNAs were sent to the Institute for Maternal and Child Health IRCCS “Burlo Garofolo” (Trieste, Italy) for the IFN signature analysis.

### Interferon-stimulated genes expression analysis

The test currently used at the Institute for Maternal and Child Health IRCCS “Burlo Garofolo” of Trieste (Italy) is based on the six-gene-set described by Crow [36].

The measure of expression of the six genes was assessed by Real-Time PCR using AB 7500 Real-Time PCR System (Applied Biosystems, USA), TaqMan Gene Expression Master Mix (Applied Biosystems, USA) and UPL Probes (Roche, Switzerland) for *IFI27*, *IFI44L*, *IFIT1*, *ISG15*, *RSAD2* and *SIGLEC1* genes. Using ABI 7500 Real-Time PCR software, each target quantity was normalized with the expression level of *G6PD* and *HPRT1*, and the relative quantification was conducted relating to a calibrator sample (mix of ten healthy controls) using the 2^−ΔΔCt^ method [37].

The median fold change of the six genes was used to calculate the “interferon score” (IFN score) for each patient. The analyses were considered as negative or positive referring to the cut-off value of 2.466 calculated by Crow to classify pathological conditions characterized by a type I IFN dysregulation [23, 24].

### Statistical analyses

Data analyses were performed by GraphPad Prism 6 software. Mann-Whitney and one-way ANOVA non-parametric test were selected to compare different groups; linear regression by calculating Spearman’s rank correlation coefficient was carried out for correlation analyses; and *p* values < 0.05 were considered significant.

### Cluster analysis

Cluster analysis was performed using the unsupervised machine learning algorithm K-means clustering [38] provided by R [39]. This analysis partitions the group into subsets characterized by similar observation provided by IFN score, SLEDAI-2K and complement mean values. Clustering results were visualized employing the R functions *fviz_cluster* (“factoextra package”) that performs the principal component analysis (PCA) and *plot3D* (“plot3D” package). Data are plotted according to both the two and the three principal components (Dim1, Dim2 and Dim3) that describe the larger part of the variance between the clusters.

## Results

### Clinics and laboratory findings

Thirty-one subjects with cSLE agreed to participate out of the forty-one recruited who met the inclusion and exclusion criteria (as described in the “Methods” section and “Study design and subjects” section). The mean age was 13.5 (range 6–18) years, 77% were girls and 39% were non-Caucasian. The mean age at diagnosis was 11.2 years (range 6–15), disease onset before puberty was 58% and mean disease duration until data collection day was 28 months (range 1–96). The median SLEDAI-2K in cSLE was 7.5 (range 0–32): five patients (16%) having high activity (≥ 11) and ten (31%) with a moderate activity (6-10).

The numerical scoring mean for the BILAG-2004 was 13 (range 0–54). Nine systems were scored from A to E: constitutional, mucocutaneous, neuropsychiatric, musculoskeletal, cardiorespiratory, gastrointestinal, ophthalmic, renal and hematologic (Table 1).

**Table 1:** British Isles Lupus Assessment Group Index-2004 (BILAG-2004) for thirty-one patients with childhood-onset SLE (cSLE)

Total, *n* = 31	A, *n* (%)	B, *n* (%)	C, *n* (%)	D, *n* (%)	E, *n* (%)
Constitutional	2 (6.4)	1 (3.2)	2 (6.4)	22 (71.0)	4 (13.0)
Mucocutaneous	1 (3.2)	5 (16.0)	4 (13.0)	20 (64.5)	1 (3.2)
Neuropsychiatric	1 (3.2)	4 (13.0)	0	8 (25.8)	18 (58.0)
Musculoskeletal	0	4 (13.0)	2 (6.4)	13 (42.0)	12 (38.7)
Cardiorespiratory	2 (6.4)	0	3 (9.7)	9 (29.0)	17 (54.8)
Gastrointestinal	1 (3.2)	0	0	4 (13)	26 (83.9)
Ophthalmic	0	0	0	1 (3.2)	30 (96.8)
Renal	6 (19.3)	5 (16.0)	5 (16.0)	11 (35.5)	4 (13.0)
Haematologic	4 (13.0)	3 (9.7)	8 (25.8)	8 (25.8)	8 (25.8)

Grade A: very active disease requiring immunosuppressive drugs and/or more than 20 mg of prednisolone or equivalent daily; grade B: moderately active disease requiring lower doses of glucocorticoids, antimalarials or nonsteroidal anti-inflammatory drugs (NSAID); grade C: mild stable disease; grade D: no current disease activity but the system had previously been affected; grade E: no current or previous disease activity

At the time of sampling, among the thirty-one patients, twelve (38.7%) presented reduced complement levels (C3 and/or C4), nineteen (61%) were positive for double-stranded DNA (anti-dsDNA) autoantibodies, fifteen (48.4%) for aPL, six (19%) for lupus anticoagulant, thirteen (42%) for anticardiolipin and five (20%) for anti-β2 glycoprotein autoantibodies. Other laboratory findings are reported in Table 2.

**Table 2:** Laboratory findings in thirty-one cSLE patients

Total, *n* = 31	Mean	Range	Reference values
Haemogram			
Haemoglobin (g/dL)	12.2	4.7–15.4	11.5–16.6
Haematocrit (%)	36.7	15.0–45.0	35.0–49.0
Leukocytes (10^3^/mcL)	5.9	2.3–10.4	4.2–14.5
Neutrophils (10^3^/mcL)	3.5	0.7–7.3	1.8–8.5
Lymphocytes (10^3^/mcL)	1.8	0.2–3.4	1.5–7.0
Platelets (10^3^/mcL)	300.6	19.0–432.0	160.0–389.0
Urine protein/creatinine ratio	0.9	0–11.0	< 0.2
ESR (mm/h)	13	2–174	< 10
CRP (mg/dL)	0.7	0–6.5	< 0.5

Reference values are reported as mean value for 6–18 years old

*ESR* erythrocyte sedimentation rate, *CRP* C-reactive protein test

The renal biopsy was performed in twenty out of thirty-one (64.5%) patients with proteinuria. According to the International Society of Nephrology/Renal Pathology Society (ISN/RPS), thirteen (65%) were class IV, five (16%) class V and two (6.4%) class IV and V. Four patients were classified as having chronic renal disease, one in peritoneal dialysis and one in haemodialysis.

The median SLICC/ACR-DI score was 0.5 (range 0–4) and ten patients had a score of 1 or higher, indicating early cumulative damage.

Twenty-six patients were using prednisone at the time of study, mean dose of 14 mg/day (range 5–40 mg/day). Immunosuppressant and/or immunomodulators had been used in twenty-two out of thirty-one patients (71%). Further therapeutic details are reported in Table 3.

**Table 3:** Therapeutic approach for thirty-one cSLE patients at the beginning of the study

Total, *n* = 31	*n* (%)
Cyclophosphamide	17 (55.0)
Azathioprine	13 (42.0)
Mycophenolate mofetil	13 (42.0)
Rituximab	3 (9.6)
Immunoglobulins	5 (16.0)
Tacrolimus	3 (9.6)
Methotrexate	1 (3.2)
Hydroxychloroquine	31 (100.0)

### Increased IFN-stimulated genes expression in patients with cSLE

**Fig. 1:**
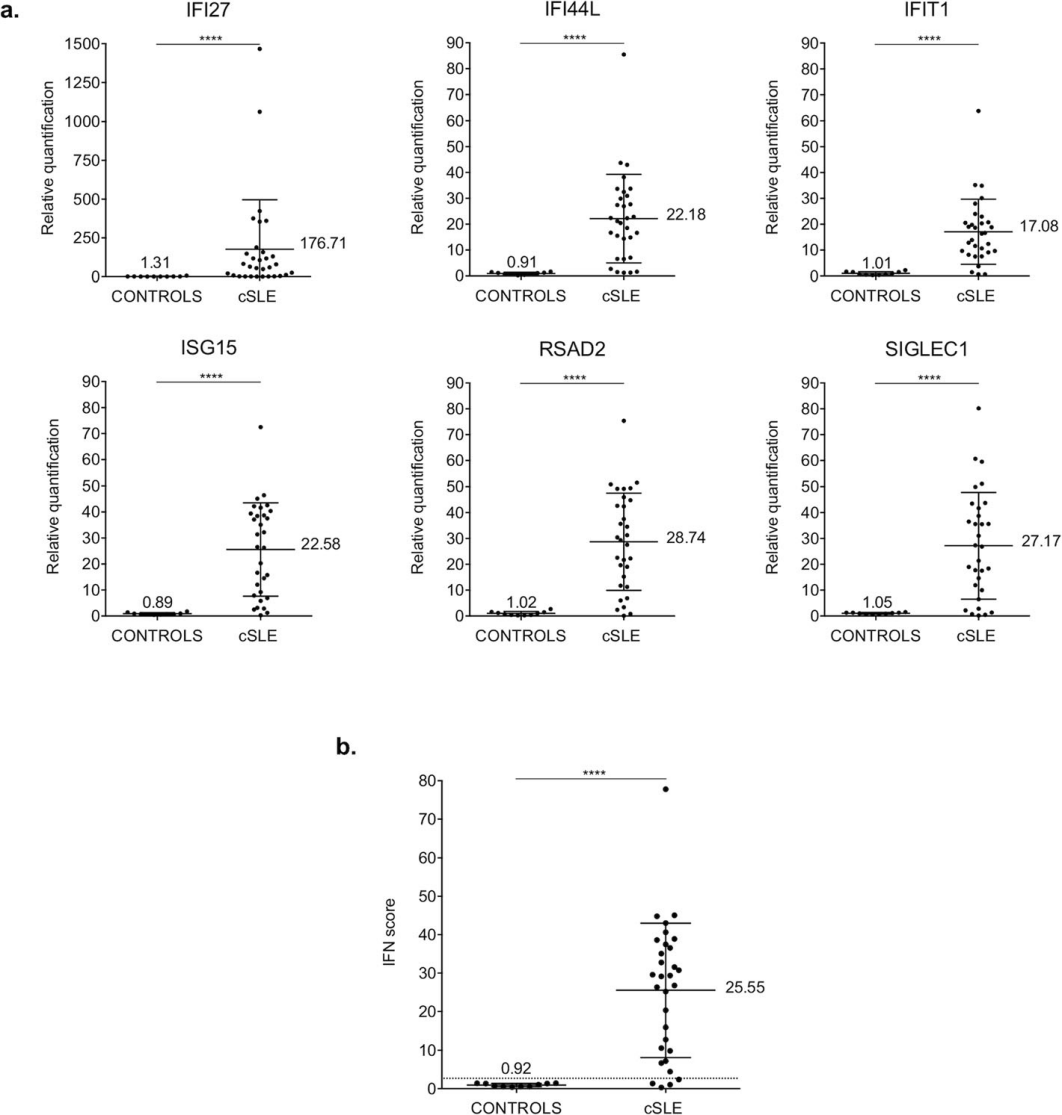
Interferon (IFN) signature analysis in thirty-one patients with childhood-onset systemic lupus erythematosus (cSLE) and ten healthy controls (CONTROLS). **a** Relative quantification (RQ) of interferon-stimulated genes (ISGs) (*IFI27*, *IFI44L*, *IFIT1*, *ISG15*, *RSAD2*, *SIGLEC1*) normalized on housekeeping genes. **b** IFN score calculated as the median of the RQ of the ISGs in patients and healthy controls. The dashed line represents the cut off value (2.466) determined by Crow [23, 36], to identify as positive/negative the IFN signature analysis. Results are reported as mean (shown in the figure) ± standard deviation. Data were analyzed with Mann-Whitney non-parametric test (****p < 0.0001)

IFN signature analysis was performed in thirty-one patients with cSLE and ten healthy controls (Fig. 1). Relative quantifications (RQ) of the six ISGs (*IFI27*, *IFI44L*, *IFIT1*, *ISG15*, *RSAD2*, *SIGLEC1*) showed significantly higher gene expression levels in patients compared with controls (Fig. 1a). The median values of the six RQ were used to calculate the IFN score for each subject (Fig. 1b). Referring to the cut-off value of 2.466 determined by Crow [23, 24], twenty-seven patients displayed a positive IFN score.

### IFN score is not correlated with disease activity

**Fig. 2:**
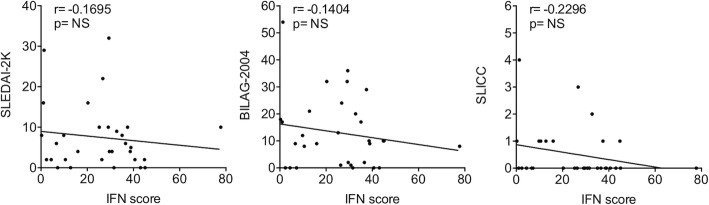
Linear regression analysis between IFN score and SLEDAI-2K, BILAG-2004 and SLICC indices by calculating Spearman’s rank correlation coefficient. *p* values < 0.05 were considered significant (NS, not significant)

Linear regression analysis was performed to investigate the correlation between the IFN score and the SLEDAI-2K, BILAG-2004 and SLICC disease activity indices, without observing any statistically significant correlation (Fig. 2).

### Patients with normal complement levels have higher IFN scores compared with patients with hypocomplementemia

Patients presenting normal complement levels had higher IFN score (*p* value = 0.04) compared with the ones with hypocomplementemia (low C3 and/or C4 levels). As expected, hypocomplementemic subjects had higher disease severity, as assessed by SLEDAI-2K (*p* value = 0.002) (Table 4).

**Table 4:** C3 and C4 levels, IFN score and SLEDAI-2K in the hypocomplementemic and normocomplementemic group

		C3 (g/L)	C4 (g/L)	IFN score	SLEDAI-2K
Reference values		0.9–1.8	0.1–0.4	Negative < 2.466	Mild < 6 Moderate 6–10 High > 11
Hypocomplementemic group	Mean	0.67	0.08	18.76	13.33
	Median	0.69	0.07	22.76	10.00
	SD	0.34	0.05	14.26	9.98
Normocomplementemic group	Mean	1.20	0.20	29.84	3.84
	Median	1.21	0.20	31.57	4.00
	SD	0.18	0.06	18.24	3.40

Data were analyzed with Mann-Whitney non-parametric test (*p < 0.05, **p < 0.01). *SD* standard deviation

### Patients with high IFN score and normal complement also display lower anti-dsDNA and may represent a “predominantly autoinflammatory” subset of cSLE

**Fig. 3:**
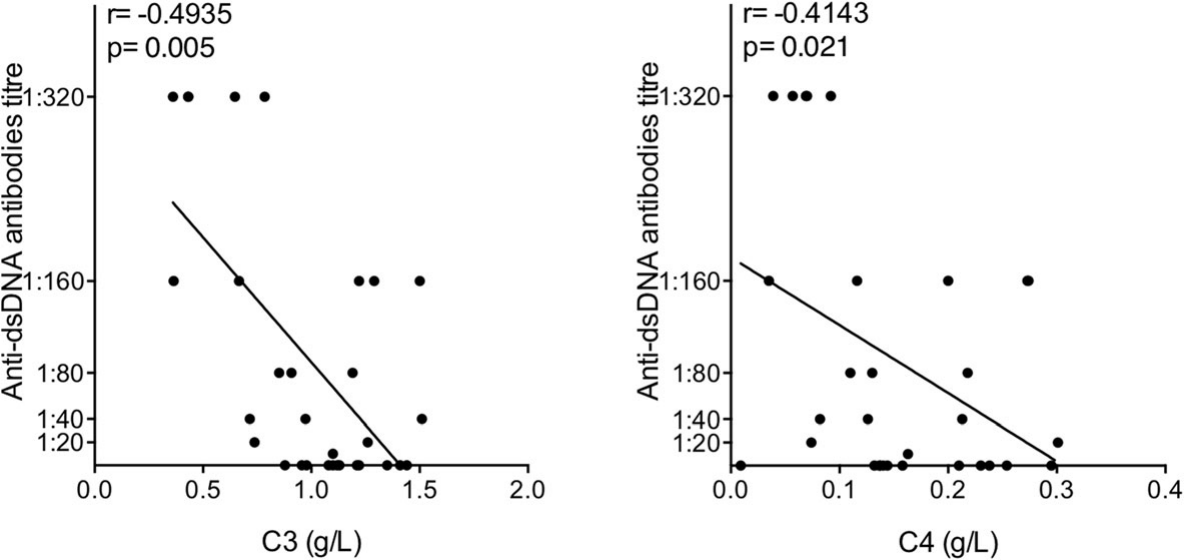
Linear regression analysis between anti-dsDNA antibodies and C3 and C4 levels, by calculating Spearman’s rank correlation coefficient. *p* values < 0.05 were considered significant

We hypothesized that the higher IFN score in patients with normal complement (Normo-C group) may identify a subgroup of cSLE characterized by a predominantly autoinflammatory component and a lower severity of autoimmune phenomena. The inverse correlation between complement levels and anti-dsDNA antibodies titre gives fuels to this interpretation. Conversely, the subgroup with hypocomplementemia (Hypo-C group) and lower IFN score is characterized by higher severity and by a predominant autoimmune component (Fig. 3).

**Fig. 4:**
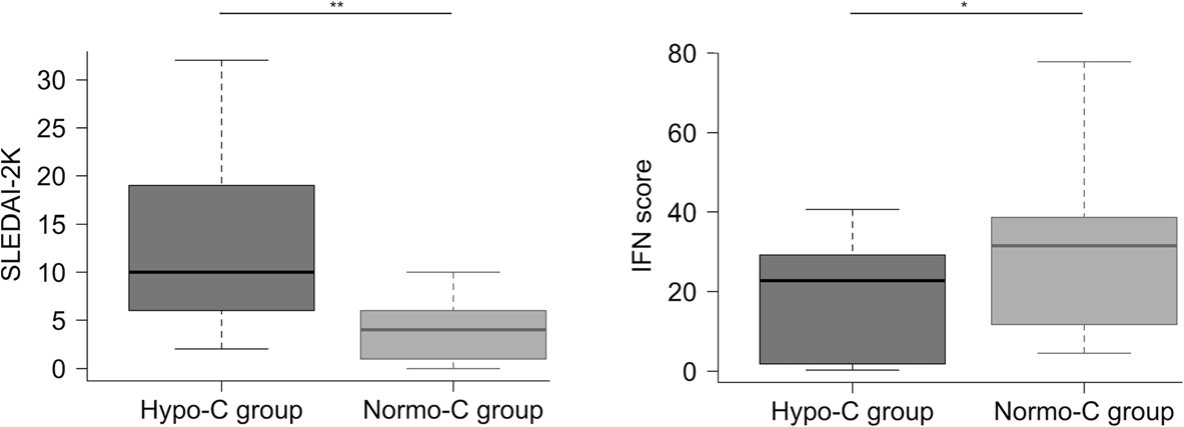
Comparison between SLEDAI-2K and IFN score values of the hypocomplementemic (Hypo-C group, *n* = 12) and normocomplementemic (Normo-C group, *n* = 19) subset. Values distribution is shown by boxplot (Whiskers 5–95 percentile). Data were analyzed with Mann-Whitney non-parametric test (*p < 0.05, **p < 0.01)

Thus, the Hypo-C group (*n* = 12 subjects) is characterized by more severe disease (higher SLEDAI-2K) and less inflammation (lower IFN score), whilst the Normo-C group (*n* = 19 subjects) presented mild disease activity (lower SLEDAI-2K) with a prominent IFN-driven inflammation (higher IFN score). The differences between SLEDAI-2K and IFN score in the two groups are shown in Fig. 4.

**Fig. 5:**
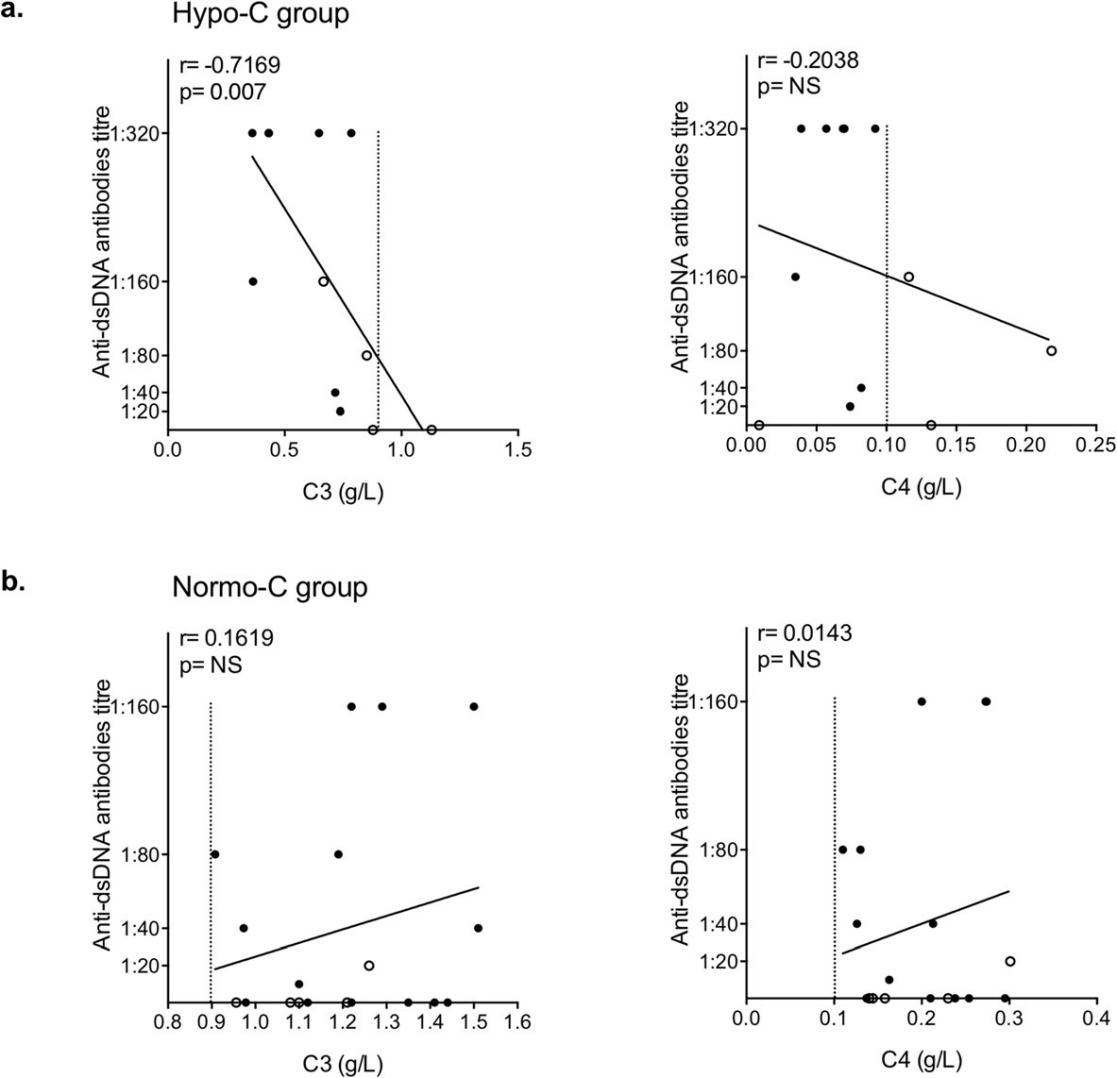
Linear regression analysis between C3 and C4 levels with anti-dsDNA antibodies in the Hypo-C (**a**) and Normo-C (**b**) group, by calculating Spearman’s rank correlation coefficient. *p* values < 0.05 were considered significant (NS, not significant). The dashed line shows the lower threshold value for C3 (0.9 g/L) and C4 (0.1 g/L). In **a**, the black dots represent patient with a low level of both C3 and C4, whilst the white dots symbolize subjects with low levels of either C3 or C4; in **b**, normocomplementemic patients which previously showed low complement levels are indicated by white dots

Notably, IFN score was even higher (mean value of 30.33) in subjects who never had low complement also in the past (represented by black dots in Fig. 5b), compared with patients with actual hypocomplementemia (mean value of 18.78).

Moreover, complement levels (C3 and C4) were analyzed in comparison with anti-dsDNA antibodies titre in the two identified groups. In the Hypo-C group (Fig. 5a), C3 levels and anti-dsDNA titre are inversely correlated, further confirming the prominent autoimmune component in these subjects. Although no significant correlation was found, also C4 levels showed an inverse correlation trend. Conversely, in the Normo-C subset of patients, there is no correlation between C3 and C4 levels and anti-dsDNA titre, and there is even a trend towards a direct correlation (Fig. 5b).

### Patients with normal complement levels display a higher IFN score than patients with hypocomplementemia, regardless of therapeutic treatments

The proposed classification of normocomplementemic patients as “predominantly autoinflammatory subset of cSLE” was reinforced by observing that the trend towards a higher IFN score in these patients was not related to the dose of anti-inflammatory drugs (prednisone and hydroxychloroquine), which did not differ significantly between the two groups. This is even more evident if we split the Normo-C group into the two subgroups of individuals who never had low complement levels (“Never Hypo-C” subgroup, *n* = 14 subjects) and those which previously showed low complement levels (“Previously Hypo-C” subgroup, *n* = 5 subjects) (Table 5).

**Table 5:** Additional information about normocomplementemic (Normo-C) and hypocomplementemic (Hypo-C) groups

			Normo-C group		Hypo-C group
			Never Hypo-C	Previously Hypo-C	
**Treatment at sample collection time**	**Prednisone (mg/day)**	Mean	15.1	2.0	19.3
		Median	7.5	0	15.0
		SD	16.9	2.7	14.4
	**Hydroxychloroquine (mg/day)**	Mean	296.4	310.0	250.0
		Median	300.0	300.0	225.0
		SD	104.6	89.4	108.7
**SLICC Damage Index**		Mean	0.4	0	0.9
		Median	0	0	0
		SD	0.5	0	1.4
**BILAG-2004**		Mean	4.1	3.0	11.9
		Median	2.5	2.0	9.5
		SD	4.8	3.3	9.8
**Age at diagnosis (years)**		Mean	12	14	10
		Median	12	14	10
		SD	3	3	2
**Age at sample collection time (years)**		Mean	14	15	13
		Median	13	15	14
		SD	3	3	4
**Disease duration (years)**		Mean	5	4	6
		Median	4	5	5
		SD	2	2	3
**Complement follow-up**	**Months**	Mean	23	21	32
		Median	15	22	20
		SD	27	13	28
	**No. of measurement (until sample collection time)**	Mean	4	4	6
		Median	3	3	6
		SD	4	2	4

The normocomplementemic (Normo-C) group has been divided into two subgroups: the “Never Hypo-C” and the “Previously Hypo-C”. Multiple comparisons were performed with Kruskal-Wallis non-parametric test. *P* values < 0.05 were considered significant (*p < 0.05). *SD* standard deviation

Noteworthy, patients who never presented hypocomplementemia and the Hypo-C patients did not display significant differences as concern prednisone (*p* value = 0.9) and hydroxychloroquine dose (*p* value = 0.8), whilst they showed the higher difference in IFN score (score 30.33 in “Never Hypo-C” vs 18.76 in Hypo-C). Conversely, subjects in the “Previously Hypo-C” subgroup, who presented an intermediate IFN score (28.46), were taking lower doses of prednisone compared with Hypo-C group (*p* value = 0.01).

Thus, we can conclude that, at least in subjects who never displayed hypocomplementemia, the higher IFN score was not related to differences in therapeutic treatments.

Lastly, since our study is cross-sectional, in most cases, the sampling did not coincide with the disease onset. Despite this remark, the Hypo-C and Normo-C subgroups did not show differences as concern age, duration of disease and damage indexes (SLICC and BILAG-2004) at the time of sample collection (Table 5). Of note, all patients underwent several measures of complement levels, allowing to well-differentiate the three subgroups.

### Cluster analysis reinforces patient classification in two different subsets

Patient subdivision has been strengthened by an unsupervised non-hierarchical cluster analysis algorithm that partitions into subgroups characterized by similar observations considering IFN score, SLEDAI-2K and mean complement levels (C3 and C4 normalized values for the respective lower threshold), analyzed separately so far.

**Fig. 6:**
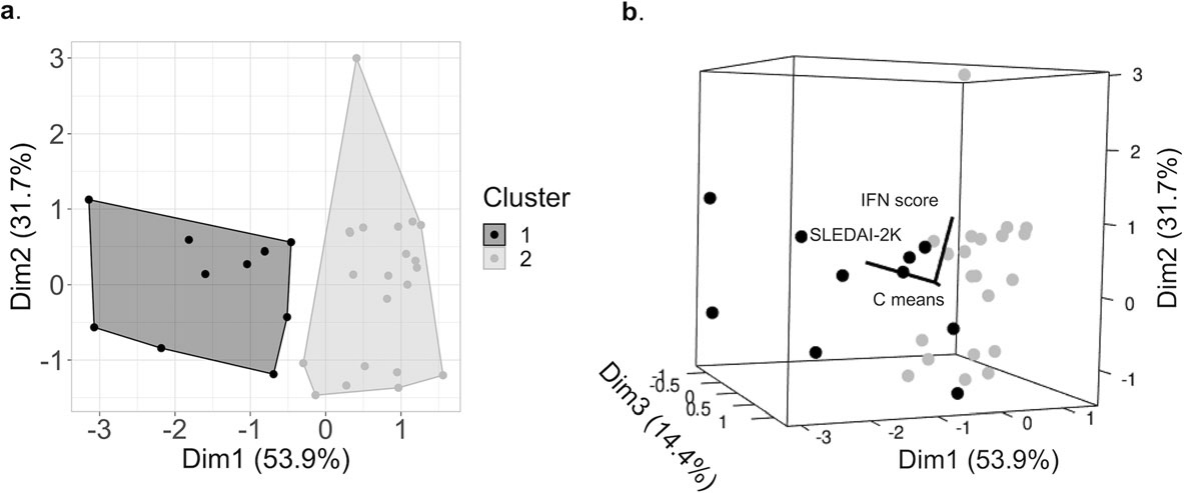
Cluster analysis results (K-means clustering) considering IFN score, SLEDAI-2K and mean complement levels (C means, mean value of C3 and C4 levels normalized on the respective lower threshold) that divide patients into subgroups by similarities. **a** 2D clusters representation (each dot represents a subject; Dim1 and Dim2 show the higher differences between the main clusters; Cluster 1: Hypo-C group, Cluster 2: Normo-C group). **b** 3D cluster representation (each sphere represents a patient; Dim3 reports minor difference in the third dimension, accentuated by SLEDAI-2K)

2D cluster (Fig. 6a) showed patient distribution into two main groups, cluster 1 (*n* = 10, Hypo-C group) and cluster 2 (*n* = 21, Normo-C group). Thus, the subgroup division is similar as determined by clinical grouping based on complement levels, except for two subjects considered as Hypo-C that have been grouped with the Normo-C subjects, due to the weakness of the algorithm to discriminate slight differences, probably introduced by border-line values of C3 and C4 levels. Data were also reported as 3D cluster plot (Fig. 6b), to better define also the slight separation of the subgroups in the third dimension accentuated by SLEDAI-2K values.

## Discussion

SLE is a complex multi-organ disease presenting both autoimmune and autoinflammatory pathogenic features, with a wide spectrum of clinical symptoms and laboratory features. Multiple genes and environmental factors are involved in the pathogenesis of the disease, influencing several biological pathways and resulting in a general dysfunction of innate and adaptive immunity.

One of the most known dysregulations occurs in the complement system, whose deficiency has been associated with more severe SLE disease activity and autoimmune response [40], especially in younger patients [41]. Recently, monogenic cases of SLE have been described in subjects with various defects in the clearance and sensing of waste nucleic acids, resulting in dysregulated type I IFN production [6, 42, 43], with seeming predominance of autoinflammatory mechanisms over autoimmune ones. Indeed, individuals with familial SLE due to dysregulated IFN inflammation may have inconstant signs of autoimmunity [12]. It is not known how these two aspects are represented in subjects with sporadic cases of SLE in children and adults.

In a pediatric series of SLE (range 6–18 years), we showed that IFN score evaluation may allow stratifying into two subgroups of the disease, characterized by the prevalence of respectively autoimmune and autoinflammatory features. Patients with normocomplementemia displayed higher IFN scores and lower disease severity as measured by SLEDAI-2K, compared with subjects with hypocomplementemia. Conversely, patients with hypocomplementemia displayed a more severe disease together with higher titre of anti-dsDNA antibodies.

The possibility that SLE can occur with normal values of complement is well known, and it is reflected by the low sensitivity of hypocomplementemia in identifying subjects with SLE [30]. However, the subgroup of patients with normocomplementemic SLE has been poorly characterized. Ramos-Casals et al. found that 62% of subjects with SLE had hypocomplementemia and that they were more likely to be females and to have nephropathy, cutaneous vasculitis and anti-dsDNA antibodies, but the research did not focus on the subgroup with normal complement values [44]. Gandino et al. showed that adults with SLE and constant normal complement levels had less frequency of haematological involvement and anti-dsDNA antibodies compared with the low-complement group, even if there was no difference in overall severity between the two groups [45]. The work from Gandino et al. did not address the intensity of IFN-mediated inflammation in the two groups and just concluded that the behaviour of complement levels is heterogeneous in SLE.

With our study, we further characterized the group of patients with normal complement values and proposed a disease model encompassing various degrees of autoinflammatory and autoimmune pathogenesis, ranging from the typical form of SLE, characterized by prominent autoimmune features, hypocomplementemia and high clinical severity, and a subset of disease with high IFN inflammation, lower autoimmunity and, at least in our experience, milder severity.

The observation that patients with normocomplementemia have higher IFN score whilst having a less severe phenotype has not been reported before, even if is not ensured that the same findings shall occur in adult population. Of note, subjects with normal complement displayed a trend towards a direct correlation between complement levels and autoantibody titres, as opposed to the typical “autoimmune” SLE, which is marked by an inverse correlation between complement and anti-dsDNA titre, supporting the interpretation that they behave as a different subset compared to hypocomplementemic SLE.

These findings may look inconsistent with data from adult-onset SLE, showing a direct correlation between lupus activity and elevated IFNα [46]. The apparent difference may be due to distinct reasons: firstly, the dosage of serum IFNα is very challenging and results were not confirmed by analysis of ISGs expression [47]; secondly, the subgroup of subjects with normal complement levels and higher IFN inflammation could be more typical and easier to detect among cSLE compared with adult-SLE.

We propose that recognizing the subgroup of SLE without hypocomplementemia and with high IFN-mediated inflammation may help patients’ stratification in future clinical trials for pediatric SLE. Whilst it could be hypothesized that drugs targeting mostly lymphocytes are appropriate to treat typical SLE, drugs targeting the IFN pathway may have a major potential in the subgroup of normocomplementemic and inflammatory SLE.

Indeed, drugs acting on the IFN pathway are increasingly used for interferonopathies treatment [48, 49]. The more listed are antimalarials, old drugs acting on the sensing of nucleic acids in lysosomes and, according to recent findings, on the activation of cGAS enzyme, the principal inducer of type I IFN signalling [50]. However, the most potent agents blocking the IFN signalling are the JAK-inhibitors, which can strongly inhibit cell response to interferons and other inflammatory cytokines [49, 51]. A phase II clinical trial recently described a possible successful employment of the JAK-inhibitor baricitinib also in subjects with SLE, but the study did not give clues to stratify patients for optimal results [52]. We believe that the possibility that subjects with “autoinflammatory” and normocomplementemic SLE may benefit much from these targeted treatments deserves attention in future trials for therapeutic stratification in pediatric SLE.

A recent study from Idborg et al. came to partly similar results by identifying two definite subgroups among patients with SLE, respectively with a profile of aPL immune response or anti-SSA/SSB (anti-Sjögren’s syndrome type A/anti-Sjögren’s syndrome type B) response. Although the authors did not focus on the group of normocomplementemic SLE, they found that the aPL-group tended to have stronger signs of autoimmunity and complement activation, whilst the SSA/SSB+ subgroup was marked by higher IFN inflammation. Thus, the authors suggested that the SSA/SSB+ subgroup may benefit from IFN-blocking therapies whilst the aPL+ subgroup is more likely to have an effect from drugs targeting the complement system [53]. Unfortunately, we could not verify the possible correlation between these groups in our series because anti-SSA/SSB antibodies values were available only for few patients.

A possible limitation in our study design could regard the sample collection time, which in most cases did not coincide with the disease onset. Indeed, few normocomplementemic subjects have presented low complement levels in the past. Although our results could be influenced by the specific moment of disease activity, the presence of a higher IFN score is confirmed ever stronger in subjects that never showed low complement levels in normocomplementemic patients regardless of therapeutic treatments.

## Conclusions

The integration between IFN signature analysis and complement levels may help distinguish two groups of subjects, which are predominantly characterized either by autoimmune or by autoinflammatory features. Prospective studies can be proposed to study if a disease stratification based on normal complement levels and increased IFN signature could be useful for therapeutic stratification in pediatric SLE.

## Data Availability

The datasets used and/or analyzed during the current study are available from the corresponding author on reasonable request.

## Abbreviations

AGS: Aicardi-Goutières syndrome
BILAG-2004: British Isles Lupus Assessment Group Index-2004
cGAS: Cyclic GMP-AMP synthase
CRP: C-reactive protein
cSLE: Childhood-onset systemic lupus erythematosus
EDTA: Ethylenediaminetetraacetic acid
ESR: Erythrocyte sedimentation rate
G6PD: Glucose-6-phosphate dehydrogenase
HIV: Human immunodeficiency virus
HPRT1: Hypoxanthine phosphoribosyltransferase 1
IFI27: Interferon-alpha inducible protein 27
IFI44L: Interferon-induced protein 44 like
IFIT1: Interferon-induced protein with tetratricopeptide repeats 1
IFN: Interferon
ISG15: Interferon-stimulated gene 15
ISGs: Interferon-stimulated genes
ISN: International Society of Nephrology
JAK: Janus kinase
NSAID: Nonsteroidal anti-inflammatory drugs
RPS: Renal Pathology Society
RQ: Relative quantification
RSAD2: Radical S-adenosyl methionine domain-containing protein 2
SIGLEC1: Sialic acid-binding Ig-like lectin 1
SLE: Systemic lupus erythematosus
SLEDAI-2K: SLE Disease Activity Index 2000
SLICC/ACR-DI: Systemic Lupus International Collaborating Clinics/American College of Rheumatology Damage Index
SSA/SSB: Anti-Sjögren’s syndrome type A/anti-Sjögren’s syndrome type B
TORCH: Toxoplasmosis, rubella, cytomegalovirus, herpes simplex
